# Distributive characteristics of the *CYP2C9* and *AGTR1* genetic polymorphisms in Han Chinese hypertensive patients: a retrospective study

**DOI:** 10.1186/s12872-021-01895-w

**Published:** 2021-02-04

**Authors:** Keping Chen, Peng Xiao, Guochun Li, Chunling Wang, Chuankun Yang

**Affiliations:** 1 Clinical Laboratory, Zhongda Hospital, School of Medicine, Southeast University, Dingjiaqiao Road 87, Nanjing, 210009 China; 2 Nanjing Central Hospital, Nanjing Municipal Government Hospital, Nanjing, 210009 China

**Keywords:** *CYP2C9*, *AGTR1*, Genetic polymorphism, Hypertension, AngiotensinIIreceptor antagonist

## Abstract

**Background:**

There is an individual variation in response to antihypertensive effect of the angiotensin II receptor antagonist. This study aimed to determine the allele and genotype frequencies of *CYP2C9* and *AGTR1* genetic polymorphisms and explore the potential role of these polymorphisms in guiding the selection of angiotensinIIreceptor antagonist in Han Chinese hypertensive patients.

**Methods:**

Totally 2419 Han Chinese hypertensive patients and 126 normotensive controls were recruited in this study. Venous blood samples were collected from each patient, and the genetic polymorphisms of *CYP2C9* and *AGTR1* were assessed using a gene chip platform. The allele and genotype frequency of each gene and the combined genotypes in this study were analyzed respectively.

**Results:**

The gene chip analysis identified an allelic frequency of 96.51% for CYP2C9*1 and 3.49% for CYP2C9*3 in the cohort of Han Chinese hypertensive patients. Statistical analysis showed that the frequency of wild-type homozygous for CYP2C9*1/*1 was 93.30%, while the frequency of heterozygous for *1/*3 or mutant homozygous for *3/*3 was 6.41% or 0.29%. Meanwhile, we detected allelic frequencies of 95.06% and 4.94% for the A and C allele of *AGTR1*, respectively. While the genotype frequency of wild-type homozygous for AA was 90.41%, the frequency of heterozygous for AC or mutant homozygous for CC was 9.30% or 0.29%. Notably, we observed that 84.66% (2048/2419) of the subjects exhibited a combined genotype of *CYP2C9* and *AGTR1* as *1/*1 + AA, while the combined genotypes *3/*3 + AC or *3/*3 + CC were not detected in hypertension patients. Besides, no significant association was found between normotensive controls and hypertensive patients, or among the three grades of hypertensive patients.

**Conclusions:**

These data revealed the polymorphisms characteristics of *CYP2C9* and *AGTR1* in Han Chinese hypertensive patients, providing valuable information for genotype-based antihypertension therapy in prospective clinical studies in the future.

## Background

Hypertension is a complex disease caused by multiple environmental and genetic factors. According to the recent nationwide hypertension survey from 2012 to 2015, the prevalence of hypertension among China adults (≥ 18 years old) is 27.9% [1]. If hypertension is not well controlled, it would inevitably become a severe public health problem in the country in the coming decades. To our knowledge, an effective way to control hypertension is to improve blood pressure management. Although hundreds of compounds representing distinct drug classes have been approved for the treatment of hypertension, the control rate of hypertension in Chinese population remains as low as 15.3% [2]. The patients with hypertension have suffered from trial–error switching of drug classes due to interindividual variations that are attributed to genetic and environmental factors [3, 4].

As a major isoform of CYP superfamily, CYP2C9 accounts for approximately 20% of the total CYP protein in the liver. Allele variants of CYP2C9 may underlie the decreased activities of CYP2C9 enzyme in the populations. Although about 30 variant alleles of *CYP2C9* have been reported, only CYP2C9*2 (Arg144Cys) and CYP2C9*3 (Ile359Leu) are well studied with respect to their reduced metabolic activities compared with the wild type counterpart CYP2C9*1 (Arg144Ile359). It has been reported that the allele frequencies of CYP2C9*2 and CYP2C9*3 range from 4 to 16% in the Caucasian populations [5], while in the Asian population, CYP2C9*2 is absent, and the frequency of CYP2C9*3 varies from 0.07 to 6.0% [6–9]. Yu et al. found that in the Chinese population, the allele frequency of CYP2C9*3 was 1.5% in hypertensive patients and 4.9% in healthy controls [10]. Losartan is a selective angiotensin II receptor antagonist and has been used in hypertension treatment to reduce the risk of cardiovascular events. As a prodrug, losartan needs to be oxidized by CYP2C9 to be an active metabolite showing the most antihypertensive effect [11]. Numerous studies reveal that CYP2C9*3 allele can reduce oxidation of losartan, thus decreasing its antihypertensive function [11, 12]. Unlike losartan, irbesartan, another angiotensin II receptor antagonist, requires CYP2C9 to be converted to inactive metabolite. Besides, the blood concentrations of irbesartan have been found to be higher in CYP2C9*3 allele carriers compared with other allele carriers [13]. Moreover, studies have shown that the plasma concentration of irbesartan in Chinese hypertensive patients carrying CYP2C9*3 allele is significantly elevated 6 h after dosing [14, 15]. Thus, CYP2C9*3 allele is involved in an individual’s response to antihypertensive drugs.

Angiotensin II is an important effector controlling blood pressure in the cardiovascular system. Type 1 angiotensin II receptor (AGTR) is encoded by the *AGTR1* gene. *AGTR1* polymorphisms are found to be associated with blood pressure response to the inhibition of renin-angiotensin system (RAS) in the hypertensive population [16, 17]. A1166C (rs5186) SNP of *AGTR1* may be involved in posttranscriptional modification and angiotensin II receptor-mediated cell signaling [18]. A1166C has been extensively studied in hypertension and is associated with the end-point phenotypes [16, 17]. Sun et al., found that in Chinese population, A1166C polymorphism of *AGTR1* was associated with antihypertensive response to candesartan, and individuals with AC genotype displayed a significantly reduction in SBP after taking candesartan [19].

It has been documented that genetic polymorphisms of *CYP2C9* and *AGTR1* are involved in the determination of the individual variation in response to antihypertensive effect of angiotensinIIreceptor antagonist. The prevalence of genetic polymorphisms varies remarkably among different geographical regions, nationalities, and races. In this study, we aimed to determine the allele frequency and genotype distribution of *CYP2C9* and *AGTR1*, and explore the relationships between hypertension and combined genotypes and phenotypes of *CYP2C9* (CYP2C9*3, Ile359Leu, rs1057910, A1075C) and *AGTR1* (rs5186, A1166C) in Han Chinese population using angiotensin II receptor antagonist. This study may provide valuable information for guiding the selection of angiotensin II receptor antagonist such as losartan, irbesartan, and candesartan.

## Methods

### Study subjects

This study was approved by the Ethics Committee of Zhongda Hospital, Southeast University. A total of 2545 Han Chinese individuals from February 2017 to December 2019 were enrolled in the retrospective study, including 2419 hypertensive patients and 126 normotensive controls. All subjects were inpatients in Zhongda Hospital. For each patient, blood pressure was measured upon admission in hospital. Hypertension was defined as systolic blood pressure > 140 mmHg and diastolic blood pressure > 90 mmHg. These patients with essential hypertensions did not take any antihypertensive drugs prior to this study. Patients with secondary hypertension such as kidney disease, endocrine diseases were excluded. Clinical characteristics of enrolled patients were shown in Table 1.

**Table 1:** Clinical characteristic of enrolled Han Chinese hypertension patients

Characteristics	Value
Age (years), mean ± SD	65 ± 14
Female, n (%)	1014 (41.92%)
Smoker, n (%)	
Never	1768 (73.09%)
Current	492 (20.34%)
Ex-smoker	159 (6.57%)
Drinker, n (%)	
Never	2069 (85.53%)
Regular drinker	350 (14.47%)
SBP (mmHg)	161 ± 15
DBP (mmHg)	97 ± 16
CHOL (mmol/L)	4.50 ± 1.17
TG (mmol/L)	1.64 ± 1.25
GLU (mmol/L)	6.51 ± 2.31
HDL (mmol/L)	1.17 ± 0.29
LDL (mmol/L)	2.78 ± 0.95
LP(a) (mmol/L)	262 ± 281
UA (μmol/L)	356 ± 113

### Genotyping procedures for *CYP2C9* and *AGTR1*

**Fig. 1:**
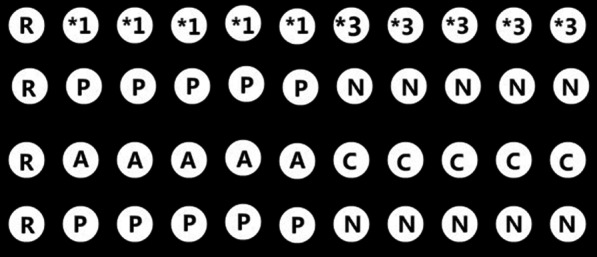
Schematic diagrams of *CYP2C9* and *AGTR1* gene chip analysis. R is the chip hybridization positioning reference. *1 and *3 are the wild type and mutant type probe sites of *CYP2C9* gene, respectively. A, C are the wild-type and mutant-type probe sites of *AGTR1* gene, respectively. P is the positive reference probe site for *CYP2C9* and *AGTR1*, while N is the negative reference probe site. There are 5 repeats for the wild-type probes, the mutant-type probes, positive reference probes, and negative reference probes

Two milliliters of venous blood were collected from each participant and stored in EDTA-containing tubes. The DNA extraction was carried out using TIANamp blood DNA kit (TIANGEN Biotechnology, Beijing, China) according to the manufacturer’s instructions. Polymerase chain reaction (PCR) was performed using the following protocol: pre-denaturation at 94 °C for 3 min, followed by 40 cycles of denaturing at 94 °C for 30 s, annealing at 56 °C for 30 s, and extension at 70 °C for 30 s, as well as a final elongation at 70 °C for 2 min. Subsequently, PCR products of *CYP2C9* or *AGTR1* were subjected to gene chip analysis, and the reaction was kept at 41 °C for 2 h. After hybridization, the gene chip was washed by three different washing buffers. Finally, GenePix4100A scanner (Molecular Devices, California, USA) was utilized for scanning. The GenePix6.0 software (www.csupharmacol.com) was employed to analyze the images of the hybridization and determine the genotypes of *CYP2C9* and *AGTR1* for each sample. All the reagents were purchased from Honghao Gene Biotechnology (Hunan, China). The schematic diagrams of the gene chip for *CYP2C9* and *AGTR1* were depicted in Fig. 1.

### Statistical analysis

Statistical Package for the Social Sciences for Windows (SPSS, version 11.0) was used for data analysis. The allele frequencies were calculated based on the observed number of the different alleles. Hardy–Weinberg equilibrium was tested using the SPSS. *p* < 0.05 value was considered statistically significant.

## Results

### The allele and genotype frequencies of *CYP2C9* and *AGTR1* polymorphisms in Han Chinese hypertensive patients

The allele frequency and genotype distribution of *CYP2C9* or *AGRT1* in the hypertensive population were shown in Tables 2 and 3, respectively. In the case of *CYP2C9*, we found an allelic frequency of 96.51% for the *1 allele and 3.49% for *3 allele. Further analysis showed that the frequency of wild-type homozygous genotype (*1/*1) was 93.30%, while the frequency of mutant heterozygotes (*1/*3) or homozygotes (*3/*3) was 6.41% or 0.29%. The *CYP2C9* polymorphism frequency was in accordance with Hardy–Weinberg equilibrium (χ^2^ = 1.81, *p* = 0.41). For the SNPs of *AGTR1*, we identified a frequency of 95.06% for A allele and 4.94% for C allele. While the frequency of AA genotype was 90.41%, the frequency of genotype AC or CC was 9.30% or 0.29%. Like *CYP2C9*, the *AGTR1* SNP frequency was essentially in agreement with Hardy–Weinberg equilibrium (χ^2^ = 0.09, *p* = 0.96).

**Table 2:** The allele frequencies of *CYP2C9* and *AGTR1*in Han Chinese hypertensive patients

Allele name	Frequency N (%)
CYP2C9*1	4669 (96.51%)
CYP2C9*3	169 (3.49%)
*AGTR1-A*	4599 (95.06%)
*AGTR1-C*	239 (4.94%)

**Table 3:** The genotype distribution of *CYP2C9* and *AGTR1* and Hardy–Weinberg equilibrium in Han Chinese hypertensive patients

Genotype	Observed number (N)	Expected number (N)	Frequency (%)	*P* value
CYP2C9*1/*1	2257	2253	93.30	0.41
CYP2C9*1/*3	155	163	6.41	
CYP2C9*3/*3	7	3	0.29	
*AGTR1-AA*	2187	2186	90.41	0.96
*AGTR1-AC*	225	227	9.30	
*AGTR1-CC*	7	6	0.29	

### The combined genotypes of *CYP2C9* and *AGTR1* in hypertensive patients

As indicated in Table 4, 84.66% (2048/2419) of the subjects displayed a combined genotype of *CYP2C9* and *AGTR1* as *1/*1 + AA. Notably, the combined genotype either *3/*3 + AC or *3/*3 + CC was not detected in these hypertensive patients because of the low frequency of the *3 allele in *CYP2C9* and the C allele in *AGTR1* SNPs.

**Table 4:** The distribution of combined genotypes of *CYP2C9* and *AGTR1* in Han Chinese hypertensive patients

*CYP2C9* genotype	*AGTR1* genotype	Reactions for β-receptors blockers		Expected efficacy	Expected adverse reactions	Frequencies
		Metabolizing capacity	Sensitivity			
*1/*1	AA	EM	Normal	+		84.66% (2048)
*1/*1	AC	EM	Slightly increased	++		8.43% (204)
*1/*1	CC	EM	Significantly increased	++		0.21% (5)
*1/*3	AA	IM	Normal	++		5.46% (132)
*1/*3	AC	IM	Slightly increased	+++		0.87% (21)
*1/*3	CC	IM	Significantly increased	+++	+	0.08% (2)
*3/*3	AA	PM	Normal	+++		0.29% (7)
*3/*3	AC	PM	Slightly increased	+++	+	0
*3/*3	CC	PM	Significantly increased	+++	++	0

*EM* extensive metabolizers, *IM* intermediate metabolizers, *PM* poor metabolizers

### The genotype distributions of *CYP2C9* and *AGTR1* in different grades of hypertension and normotensive controls

To examine the genotype distributions of *CYP2C9* and *AGTR1* among different grades of hypertension, we divided all hypertensive patients into three different grades based on the blood pressure levels. The criteria for each grade were defined as follows: grade 1 (systolic blood pressure 140–159 mmHg or diastolic blood pressure 90–99 mmHg); grade 2 (systolic blood pressure 160–179 mmHg or diastolic blood pressure 100–109 mmHg); and grade 3 (systolic blood pressure ≥ 180 mmHg or diastolic blood pressure ≥ 110 mmHg). As shown in Table 5, there was no significant difference in the distributions of *CYP2C9* and *AGTR1* genotypes between normotensives and hypertensive patients, or among all three grades of hypertensive patients.

**Table 5:** The genotype distributions of *CYP2C9* and *AGTR1* in different grades of Han Chinese hypertensive patients and normotensive controls

Genotype	Normotensive % (N)	The grades of hypertension % (N)			*p* value
		Grade 1	Grade 2	Grade 3	
CYP2C9*1/*1	96.03% (121)	96.43% (135)	94.13% (577)	92.74% (1545)	0.44^a^ 0.48^b^
CYP2C9*1/*3	3.97% (5)	3.57% (5)	5.71% (35)	6.90% (115)	
CYP2C9*3/*3	0.00% (0)	0.00% (0)	0.16% (1)	0.36% (6)	
*AGTR1-AA*	92.06% (116)	87.86% (123)	88.74% (544)	91.24% (1520)	0.20 ^a^ 0.19 ^b^
*AGTR1-AC*	7.14% (9)	12.14% (17)	11.09% (68)	8.40% (140)	
*AGTR1-CC*	0.79% (1)	0.00% (0)	0.16% (1)	0.36% (6)	

^a^*p* value between normotensives and hypertensive patients

^b^*p* value among all three grades of hypertensive patients

## Discussion

This study showed that the frequency of CYP2C9*3 allele was 3.49%, and CYP2C9*1/*1 was the major *CYP2C9* genotype with a frequency of 93.30% in Han hypertensive patients. Importantly, a genotype frequency of 0.29% for mutant-type homozygotes CYP2C9*3/*3 was noticed in the hypertensive patients, whereas CYP2C9*3/*3 genotype was not detected in the normotensive Han population. On the contrary, one recent research has indicated that the frequency of CYP2C9*3/*3 is 0.1% in the normotensive Han population [20]. These inconsistent conclusions may be due to different samples sizes. The present study had large hypertensive population and small normotensive population. Hiltunen et al. have stated that, small sample size has insufficient power to detect a SNP with minor allele frequency [21].

In addition, the frequency of AA genotype was 90.41% and that of CC genotype of *AGTR1* was 0.29% in Han Chinese hypertension patients. Previous studies report a frequency of 93.4% or 89.6% for the AA genotype in the Han hypertensive population [19, 22]. Collectively, these results indicated that the AA genotype of *AGTR1* was common in Han hypertensive patients.

In this study, we further identified *1/*1 + AA as the most frequent genotype of combined *CYP2C9* and *AGTR1* in Han hypertensive population. There was no significant association between the genotype distribution of *CYP2C9* or *AGTR1* and hypertension grade in Han hypertensive patients. Besides, no significant difference in the genotype distribution and allele frequency of *AGTR1* and *CYP2C9* was detected between Han normotensives and hypertensive patients. Under strictly controlled conditions, Hiltunen et al. do not identify the association between *AGTR1* and *CYP2C9* polymorphisms and antihypertensive effect of angiotensin receptor antagonists by using GWAS technology. However, they find that the SNPs of *LRPPRC, PPM1B* and *NPHS1* are significantly associated with the response to angiotensin receptor antagonists. Hypertension is a multifactorial disorder, and various parameters such as age, ethnicity, disease type, and gene–gene interactions can affect the antihypertensive response. Different ethnic group may contribute to these contradictory results. Of the top 20 SNPs associated with losartan response in GENRES study, few SNPs could be replicated in other 4 studies in Hiltunen et al. research [21], which reconfirm the complexity nature of the antihypertensive response.

Several researches have clarified that the *AGTR1* and *CYP2C9* polymorphisms are associated with the antihypertensive function of angiotensin II receptor antagonists [11–15, 18, 19]. Angiotensin II receptor antagonists are mainly metabolized by CYP2C9, and AGTR1 is implicated in the sensitivity of angiotensin II receptor antagonists. Based on these clinical studies and the present results, we speculated that most the Han Chinese hypertensive patients had normal clearance (irbesartan) or activation (losartan) function, and normal sensitivity for angiotensin II receptor antagonist. Because of the multigenic and multifactorial nature of the antihypertensive drug response, and sometimes contradictory results, prospective clinical researches are required to establish reliable recommendations.

### Limitations

The current study provided valuable information about personalized blood pressure control, but a few limitations should be considered. First, only dominant variants of *CYP2C9* (rs1057910, A1075C) and *AGTR1* (rs5186, A1166C) were detected because of their high frequencies in Han Chinese. Second, there were a large number of hypertensive patients in the present study, but the sample size of normotensive controls was relatively small. Finally, the pharmacokinetics of angiotensin II receptor antagonist among individuals with different *CYP2C9* and *AGTR1* genotypes were not evaluated.

## Conclusions

In summary, this study reveals the polymorphisms characteristics of *CYP2C9* and *AGTR1* in Han Chinese hypertensive patients. CYP2C9*1/*1 and *AGTR1-AA* are the major genotypes, and *1/*1 + AA is the most frequent genotype of combined *CYP2C9* and *AGTR1*. Additionally, no significant differences in genotype distributions are revealed between normotensives and hypertensive patients, or among all three grades of hypertensive patients. Further prospective clinical studies in Han hypertensive patients are required to establish reliable therapy recommendations.

## Data Availability

The datasets analyzed during the current study are available from the corresponding author on reasonable request.

## Abbreviations

CYP2C9: Cytochrome P450 2C9
AGTR: AngiotensinIIreceptor
RAS: Renin-angiotensin system
PCR: Polymerase chain reaction
SBP: Systolic blood pressure
DBP: Diastolic blood pressure
CHOL: Cholesterol
TG: Triglycerides
GLU: Glucose
HDL: High density lipoprotein
LDL: Low density lipoprotein
LPA: Lipoprotein (a)
UA: Uric acid
